# Aflatoxins Contamination in Maize Products from Rural Communities in San Luis Potosi, Mexico

**DOI:** 10.29024/aogh.918

**Published:** 2018-07-27

**Authors:** Beatriz A. Zuki-Orozco, Lilia E. Batres-Esquivel, María D. Ortiz-Pérez, Bertha I. Juárez-Flores, Fernando Díaz-Barriga

**Affiliations:** 1 Coordinación para la Innovación y Aplicación de la Ciencia y Tecnología, Universidad Autónoma de San Luis Potosí, 78210 San Luis Potosí, MX; 2 Instituto de Investigación en Zonas Desérticas, Universidad Autónoma de San Luis Potosí, MX

## Abstract

**Background:**

Aflatoxins are a group of mycotoxins that have been associated with hepatic damage and cancer. Aflatoxins B1 and B2 are secondary metabolites produced by fungi Aspergillus. These toxins can be found in a variety of commodities, especially in maize, and have been studied around the world due to their effects in human health. The Latin American population is especially exposed to aflatoxins given that maize products can be found in traditional diets all over the continent. Interestingly, in Mexico, chronic hepatic diseases and cirrhosis are leading causes of death in adult population.

**Methods:**

In order to observe the effect of physical variables like temperature and humidity, this study was conducted collecting samples in four different seasons, in two communities in the State of San Luis Potosi, in Mexico. The content of aflatoxins in tortillas was measured using immunoaffinity columns prior to HPLC-FLD analysis.

**Findings:**

Results showed that 18% of samples exceeded the Mexican limits for AFB1; whereas, 26% of the samples exceeded the limits of the European Union for AFB1. The AFB1 was detected in 80% of samples in one site and higher concentrations were found in samples collected during fall and winter seasons.

**Conclusions:**

Lack of control in storing practices is the principal cause for the contamination of maize. Considering that maize products are part of the staple diet of Mexican population, our results show that AFB1 detection has to be declared a public health priority. Detection and prevention of aflatoxins through a surveillance program, may avoid chronic health effects.

## Introduction

Aflatoxins are mycotoxins produced mainly by *Aspergillus flavus* and *A. parasiticus* [1]. The first species is known to produce only aflatoxin B (AFB_1_ and AFB_2_), while the second one produces both aflatoxins B and G. *A. flavus* is widely distributed around the world, but it is more likely to develop in tropical zones. These fungi can infect maize, peanuts, cotton seeds and a variety of crops and dried products.

Aflatoxins are among the most genotoxic natural products. AFB_1_ is hepatotoxic in animals and humans [1,2]. Furthermore, immune function in children [3,4] and nephrotoxic effects in animals [1,5] have also been reported. AFB_1_ increases the mitochondria permeability and it causes alterations in RNA metabolism [6]; in addition, the mechanism for cancer development due to aflatoxin B_1_ involves the activation of a genotoxic epoxide, generation of DNA adducts, as well as modifications in the TP53 gene [2]. AFB_1_ and a mixture of aflatoxins containing it, are listed in group I by the IARC [2] as they have been considered carcinogenic agents in humans.

Worldwide, it has been estimated that the people at risk of exposure to aflatoxins is between 4.5 and 5.5 billion [7,8]. This is an important number considering how epidemiological studies have shown a correlation between aflatoxin exposure and hepatocellular carcinoma (HCC) [9]. This result is a public health issue, since in developing countries, HCC is one of the leading causes of cancer deaths [10]. Interestingly, the combination of chronic exposure to aflatoxins and Hepatitis B virus (HBV) infection lead to an increased risk of cancer when compared to individuals only exposed to aflatoxins [8,11]. Another study demonstrated roughly a sixtyfold increase of risk of HCC in patients exposed to aflatoxins and chronic HBV. A synergistic effect of aflatoxins and Hepatitis C virus has also been reported [12].

In Mexico, official health reports show that chronic hepatic diseases and cirrhosis are the fifth leading cause of death in the general population, increasing its incidence in the adult population above 35 years old [13]. Multiple factors like food and alcohol ingestion, blood transfusions and surgery can contribute to the development of liver diseases. Though the main cause reported for liver cirrhosis is alcohol consumption, viral etiology and others need to be addressed [14]. Regarding neoplasms, liver cancer is listed as one of the top six of causes of death in 2016 [15].

In this context, the exposure to aflatoxins has to be considered a risk factor involved in hepatic diseases in Mexico, as these toxins have been found in some components of the Mexican diet.

The consumption *per capita* of maize per year in Mexico is estimated to be 120 kg [16], while the *tortilla* consumption per day is estimated to be 155.4 g in urban areas [17] and 217.9 g in rural zones [18]. Despite its occurrence in a variety of products, only milk, dough, flour, *tostadas* and tortillas are regulated by two guidelines of the Ministry of Health: the NOM-187-SSA1/SCFI-2002 and NOM-188-SSA12-002 [19,20]. The maximum allowed level for AFB_1_ is 20 μg/kg (ppb), in contrast to the level allowed by the European Commission regulation, which is 5 μg/kg [21].

The most significant event of aflatoxin contamination of maize in Mexico took place in the state of Tamaulipas in 1989. The combination of high temperature and drought favored the development of plagues, and therefore the presence of aflatoxins, in concentrations about of 456–5 µg of AFB_1_/kg and of 250 µg/kg after storage [22,23]. Since then, the presence of aflatoxins in maize has been studied in maize as well as in tortillas [24,25]. In this regard, a study of aflatoxins in tortillas in Mexico City shows that 20% of the analyzed *tortillas* exceeded the levels established by the Mexican regulation and almost 70% of them with AFB_1_ at a mean level of 12.1 µg/kg [26]. Asimilar work reported a mean aflatoxin concentration of 28.5 µg/kg [27].

Food preparation influences the content of aflatoxin in tortillas, though they are not quickly degraded during cooking [28,29]. For example “*nixtamalizacion*” which is a process of cooking kernels with calcium hydroxide [30], can reduce aflatoxins concentration in tortillas [19,20,21].

Despite the evidence, a surveillance program regarding exposure to aflatoxins and food safety for prevention is still needed. This program has to take into account, not only aflatoxin concentrations in maize products, but also factors such as the elaboration process, origin of maize, weather and handling conditions, etc., as they have effects on the final aflatoxin content.

Therefore, the present study had two main objectives. The first one was the validation of an analytical method, using immunoaffinity columns and a post-column UV photo derivatization to determine low concentrations of aflatoxins in tortillas; and the second one was the measurement of aflatoxins in tortillas from two communities in San Luis Potosi, Mexico, in four different sampling periods to account for climate variations.

## Materials and Methods

The material used for setting the method consisted of tortillas obtained from different sources including local markets, tortilla shops and supermarkets. Calibration curves and method performance were executed with a homogenized stock containing 2.5 g of tortillas that were spiked by adding different concentrations of aflatoxin standard solutions by triplicate to obtain a calibration graph with concentrations of: 1.5, 3.0, 6.0, 12.0 and 15.0 μg/kg. A blank was also prepared. Additionally, another three graphs were prepared in different days to have a total of six graphs for calculating limit of quantification (LOQ). The calculated method recovery of AFB_1_ standard solution was 92%, agreeing with the value reported by the manufacturer.

The processing of the samples was carried out with 2.5 g of the dry sample. To decrease the volume of solvent and therefore, the waste generated, the volume was reduced to only 8 mL of the extraction solution (acetonitrile: water 85:15 v/v). After that, it was mixed for 60 minutes in an oscillator at 37°C. The extract was evaporated at 45°C with a stream of nitrogen and then diluted with a 0.1 M phosphate buffer solution to reduce the acetonitrile volume to 5% of the total volume. For the clean-up, we used immunoaffinity columns AflaStar™ R (Romer Labs) following the manufacturer’s procedure. Then the columns were eluted with three volumes of 1 mL of HPLC grade methanol and the resulting volume was evaporated to dryness at 45°C under a stream of nitrogen and reconstituted in 1 mL of mobile phase (water: methanol 65:35 v/v) for analysis.

HPLC analyses were carried out with an Agilent 1260 Infinity LC System coupled with FLD, including a C_18_ Poroshell 120 analytical column (4.6 × 50 mm, 2.7 µm). The operational conditions of the FLD were optimized at 362 nm for excitation, and both 440 and 460 nm for emission. The mobile phase consisted of water: methanol (65:35) at a flow of 0.8 mL/min. The column temperature was set at 40°C and the derivatization was carried out by the UVE *Post column UV derivatization* module, from LCTech.

After the optimization of the described method, the analysis of aflatoxins in tortillas coming from two rural communities was performed. The *tortillas* were collected in 2015 in two communities of San Luis Potosi: Tocoy, a small indigenous community located in the Huasteca zone in San Antonio; and Estacion Bocas, a suburban community located in the central zone of the state.

Tocoy is a rural community with an average annual temperature of 222–6°C and abundant rainfall during summer [31]. In this indigenous community, tortillas are prepared in a traditional way and individually in every home, since there is no local place for commercial *tortilla* production like in the urban areas. Depending on the season, some families elaborate the tortillas and other maize products from their own harvest. The rest of the year, the maize is provided by local markets and mainly by Diconsa, which is a national food supplier for marginal communities in Mexico. Estación Bocas is a peri-urban site with rural practices, that has an average annual temperature of 16.8°C and dry climate. In contrast with Tocoy, most families buy tortillas from three local tortilla shops; however, as in Tocoy, the maize used in the shops is distributed by Diconsa. Only few people in Bocas have their own harvested maize. For this reason, the number of samples in this community was smaller compared to those from Tocoy. Tortilla samples were obtained in four periods in both communities, in February–March, June, September and December.

The *tortillas* were donated by 45 and 49 families in Tocoy and E. Bocas, respectively, resulting in a dry weight of 12 to 18 g per sample. They were received in plastic bags and preserved at –20°C until the moment of analysis. Then, they were dried and grinded as described in the validation procedure. Participants were asked to answer a survey/questionnaire about the origin of the maize used to make tortillas, consumption, preparation practices and storage conditions.

By the time we submitted the results, we called for a meeting to explain the aim of the study, the report content and the risks we found. Subsequently, there was a second meeting with the objective to make a workshop in which everyone could participate by giving examples of storage practices, discuss about contamination problems, harvesting procedures and post-harvest handling.

## Results and Discussion

Aflatoxin levels were calculated on a dry weight basis. The LOD and LOQ were calculated from data obtained from eight graphs [32]. The method performance is shown in Table 1. Aflatoxin concentrations were calculated with a LC of 95%. The trueness result from the certified reference material (ERM®) was of 100.4% for AFB_1_ and 98% for AFB_2_. Values obtained of AFB_2_ were under the LOD or non-detectable, so statistical analysis was performed only on the AFB_1_ data.

**Table 1:** Method performance for AFB_1_, AFB_2_.

Aflatoxin	LOD (µg/kg)	LOQ (µg/kg)	r

AFB_1_	0.102	0.186	0.999
AFB_2_	0.009	0.017	0.999

R = Correlation Coefficient. LOD = Limit of Detection LOQ = Limit of Quantification.

In Table 2, it can be observed that 61% of samples from Tocoy and 27% from Bocas were above the LOQ for AFB_1_. The highest percentage of samples in Tocoy above the LOD (more than 90%) and higher than the Mexican or European regulations were found in those collected during December. However, the highest concentration found belongs to the third sampling period (September). In E. Bocas, the highest AFB_1_ concentration belongs to the first sampling period.

**Table 2:** Descriptive statistics of AFB_1_ concentration by site, in µg/kg.

Site	n	Median	Max	>LOD%	>LOQ%	% > Mx	% > EU

Tocoy	123	0.172	287.230	81	61	9	14
P1	18	0.051	1.391	50	20	0	0
P2	31	0.051	9.567	53	29	0	3
P3	28	0.069	287.230	61	39	13	19
P4	36	1.707	255.545	92	89	18	26
E. Bocas	48	0.008	19.019	54	27	6	8
P1	27	0.051	19.019	48	23	6	10
P2	9	0.051	0.433	58	17	0	0
P3	6	0.293	15.173	80	80	20	20

P1 = February; P2 = June; P3 = September; P4 = December. >LOD = Above Limit of Detection; >LOQ = Above Limit of Quantification; Mx = Mexican maximum level allowed for total AFs: 12 µg/kg (NOM-187-SSA1/SCFI-2002). EU = European Union maximum level allowed for total AFs: 5 µg/kg AFB_1_ [21].

When compared to the European Union regulation, 14% of the total tortillas from Tocoy exceeded the AFB_1_ limit, while only 8% of samples from E. Bocas did (Table 2). The results are of interest considering that the average consumption of tortillas in the indigenous region is three times higher than that calculated for urban population.

Results from AFB_2_ concentrations are presented in Table 3. Only a small number of samples could be quantified compared to the AFB_1_, except for the last sampling period in Tocoy.

**Table 3:** Descriptive statistics of AFB_2_ concentration by site, in µg/kg.

Site	n	Median	Max	>LOD%	>LOQ%

Tocoy	123	0.004	30.700	29	28
P1	9	0.004	0.122	5	5
P2	19	0.004	0.536	17.6	18
P3	20	0.004	16.911	19.3	19
P4	36	0.149	30.700	60	58
E. Bocas	48	0.503	0.927	10	10
P1	11	0.503	0.927	13	13
P2	5	0.004	0.004	0	0
P3	5	0.004	1.068	20	20

P1 = February; P2 = June; P3 = September; P4 = December. >LOD = Above Limit of Detection; >LOQ = Above Limit of Quantification.

**Figure 1:**
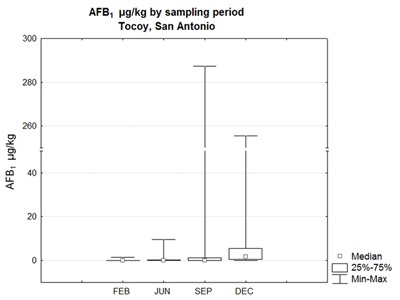
Median values of AFB_1_ concentrations in samples from Tocoy, reported in µg/kg, obtained in February, June, September and December of 2015.* Statistical difference in December (p < 0.0005).

Data dispersion was processed by the Shapiro–Wilk test, observing a non-normal distribution. A non-parametric Kruskal-Wallis test showed a statistical difference between the total AFB_1_ concentrations of the two sites (p = 0.0054). The test was also applied to the data grouped by sampling period in each site, observing significant difference only in Tocoy, where the last sampling period (December) was statistically different from the others (Figure 1).

**Figure 2:**
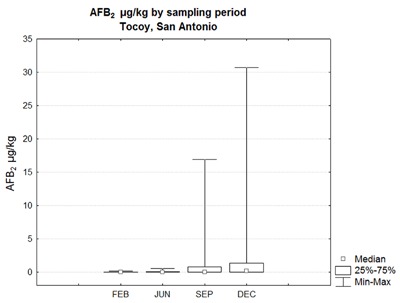
AFB_2_ concentration in samples from Tocoy, reported in µg/kg, obtained in February, June, September and December of 2015. No statistical difference in sampling groups (p = 0.01).

For AFB_2_, no significant differences between groups in both sites were found; interestingly however, dispersion of the data was higher in September and December (Figure 2).

Another Kruskal-Wallis test was applied to the AFB_1_ concentrations by source of maize in Tocoy, since 50% of the families had harvested maize, while the rest bought it from local stores. The test results showed no statistical differences, which made us focus more on the influence that physical conditions have over the fungal growth and aflatoxin production.

**Figure 3:**
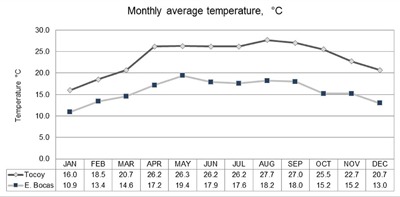
Average monthly temperature in Tocoy and Estacion Bocas, San Luis Potosi. Conagua, 2015.

Considering that AFB_1_ concentrations in samples collected in Tocoy during December couldn’t be explained by the source of maize, we proceeded to analyze the influence of temperature on the aflatoxin concentration. In Figure 3 monthly average temperatures are shown, and as expected, lower temperatures can be observed during winter. In terms of storage conditions, lower temperature can protect from fungal infection, but increases water condensation, representing a significant risk to plagues and fungal proliferation. Therefore, February AFB_1_ concentrations should also be high, but they are not. Thus, among others, factors such as variations in preparation procedures and water content during harvest have to be taken into account in order to explain December AFB_1_ concentrations in *tortillas*.

In this context, surveillance of maize constitutes an issue that should be considered a priority since it is currently out of the reach of government agencies. Only large companies perform maize analyses and there are not enough resources to ensure the quality and safety of maize consumed under traditional practices.

The high consumption of tortilla and other maize products, as well as other food products containing aflatoxins, constitutes a chronic risk for developing hepatic diseases and cancer for Mexican population. Nevertheless, Latin American countries also share a high maize consumption tradition, and, as showed in Table 4, the presence of aflatoxins has been observed in other studies outside Mexico.

**Table 4:** Aflatoxins detected in maize, Latin America.

Country	Sample	AF’s concentration

Mexico (1995) [24]	Maize	73 µg/kg^a^
Mexico (2011) [26]	Maize tortillas	0.003–0.385 µg/kg
Guatemala (1988) [33]	Maize cake	51 µg/kg^a^
	Maize	<4 µg/kg^a^
Cuba [34]	Maize	109–5 µg/kg
Costa Rica [35]	Maize	50 µg/kg^a^

^a^Mean value.

As shown in Tables, aflatoxins were present in *tortillas* from Tocoy and E. Bocas in different sampling periods. The highest concentrations of AFB_1_ and AFB_2_ were found in Tocoy, where we could observe that the last sampling period (December) showed the highest levels of AFB_1_.

Given that weather and source of maize were not the decisive factors to explain that occurrence, it is important to conduct further research regarding variables to take into consideration, like origin of maize, storage conditions, temperature, humidity and handling.

The results presented in this study showed the presence of aflatoxins in two sites regardless of the geographical, cultural, ethnic and economic differences. This occurrence tells us about the relevance of the problem, showing that contamination of maize with aflatoxins is not limited to a zone or a season. Considering the importance of maize as a basic dietary component and the risk of contamination across the country and throughout the year, the need to think of applicable strategies for control and prevention is evident. For that purpose, it is necessary to create a national surveillance program for maize, including not only food analysis but actions directed to control and prevention. According to observations obtained in this study, each community has its own risk factors that must be addressed in a local way to understand their conditions towards getting favorable results. In this regard, analytical methods should be improved to increase their economical accessibility.

The current research addressed only the aflatoxin occurrence, but microorganisms and insects can cause a variety of pre- and post-harvesting problems, leading to food loss and chronic health issues. Fumonisins, Ochratoxin A and Zearalenone are mycotoxins known as serious threats to prenatal health, kidney disease and endocrine disruptors respectively; therefore, good practices can prevent not only aflatoxins presence but synergistic effects with these and other mycotoxins.
